# Activation of innate antiviral immune response via double-stranded RNA-dependent RLR receptor-mediated necroptosis

**DOI:** 10.1038/srep22550

**Published:** 2016-03-03

**Authors:** Wei Wang, Wei-Hua Wang, Kazem M. Azadzoi, Ning Su, Peng Dai, Jianbin Sun, Qin Wang, Ping Liang, Wentao Zhang, Xiaoying Lei, Zhen Yan, Jing-Hua Yang

**Affiliations:** 1 The State Key Laboratory of Cancer Biology, Department of Pharmacogenomics, School of Pharmacy, The Fourth Military Medical University, Xi’an, 710032, China; 2 Departments of Surgery and Urology, VA Boston Healthcare System, Boston University School of Medicine, Boston 510660, MA, USA; 3 Departments of Neurosurgery and Oncology, Xijing and Tangdu Hospital, Xi’an, China; 4 Cancer Research Center, Shandong University School of Medicine, Jinan, 250012, China

## Abstract

Viruses induce double-stranded RNA (dsRNA) in the host cells. The mammalian system has developed dsRNA-dependent recognition receptors such as RLRs that recognize the long stretches of dsRNA as PAMPs to activate interferon-mediated antiviral pathways and apoptosis in severe infection. Here we report an efficient antiviral immune response through dsRNA-dependent RLR receptor-mediated necroptosis against infections from different classes of viruses. We demonstrated that virus-infected A549 cells were efficiently killed in the presence of a chimeric RLR receptor, dsCARE. It measurably suppressed the interferon antiviral pathway but promoted IL-1β production. Canonical cell death analysis by morphologic assessment, phosphatidylserine exposure, caspase cleavage and chemical inhibition excluded the involvement of apoptosis and consistently suggested RLR receptor-mediated necroptosis as the underlying mechanism of infected cell death. The necroptotic pathway was augmented by the formation of RIP1-RIP3 necrosome, recruitment of MLKL protein and the activation of cathepsin D. Contributing roles of RIP1 and RIP3 were confirmed by gene knockdown. Furthermore, the necroptosis inhibitor necrostatin-1 but not the pan-caspase inhibitor zVAD impeded dsCARE-dependent infected cell death. Our data provides compelling evidence that the chimeric RLR receptor shifts the common interferon antiviral responses of infected cells to necroptosis and leads to rapid death of the virus-infected cells. This mechanism could be targeted as an efficient antiviral strategy.

Since life is originated from the RNA World^1^,^2^,^3^, it is postulated that double-stranded RNA (dsRNA) may be the earliest form of life. Indeed, dsRNA has been documented in many species like viruses and worms as well as plants as an essential genetic and functional constituent. In mammals, however, the long stretch of dsRNA has become a “dark matter of genome” that is not readily detected under healthy conditions^4^. It has become a common trait that most viruses, if not all, induce double-stranded RNA in mammalian cells, perhaps as the intermediates of virus replication and/or inducible transcripts of cell origins such as *Alu* RNAs^5^,^6^. For that reason, the long stretch of dsRNA has evolved as an endogenous danger signal or pathogen-associated molecular pattern (PAMP) that is required for the mammalian systems to provoke dsRNA-dependent antiviral innate immunity^7^.

Toll-like receptor (TLR) is one of the extensively investigated families of pathogen recognition receptors (PRRs), of which, TLR3 is known to encounter viral dsRNA in the endosome where viruses enter through the endocytic pathway or by uptake of the apoptotic bodies from virus infected cells. As a dsRNA PRR,TLR3 senses dsRNA and initiates type I interferon (IFN-α, β) signaling pathway via a Toll/interleukin-1 receptor (TIR)-domain-containing adapter-inducing interferon-β (TRIF) signal, which activates transcription factor interferon receptor factor 3 (IRF-3) and nuclear factor κB (NF-κB), leading to IFN-β expression^8^. It is postulated that TLR3 critically affects the induction of adaptive immunity by initiating cross-priming of T cells and mediating NK activation^9^.

Some of the striking discoveries over the past 10 years relate to the exploration of intracellular dsRNA PRRs, retinoic acid-inducible gene 1 (RIG-I) and melanoma differentiation-associated protein (MDA5) known as RIG-I-like receptors (RLRs), that are shown to detect dsRNA and provoke innate antiviral responses^7^. The RIG-I-like receptors, consisting of the dsRNA binding domain (dsRBD) and the caspase activation and recruitment domain (CARD) represent a family of natural dsRNA-dependent CARD-containing PRRs primarily regulating inflammatory responses and apoptosis during viral infection^7^,^10^,^11^. These dsRNA receptors are shown to recruit mitochondrial antiviral-signaling protein (MAVS, also known as VISA/Cardif/IPS-1) to activate TANK-binding kinase 1/inducible IκB kinase (TBK1/IKKi) and IKK complex^12^. As a result, phosphorylated IRF-3 homo- or hetro-dimerizes with IRF-7, to induce type I interferon expression^13^, and eventually initiate programmed cell death (PCD) in host cells, usually via apoptosis mechanism, to eliminate severely infected cells.

Unlike TLR3, CARD-CARD interaction is critical for RIG-I-like receptors to recruit downstream CARD-containing signal transducers to initiate the antiviral responses^12^. Moreover, for well-characterized inflammatory responses, NOD-like receptors (NLRs) recruit downstream apoptosis-associated speck-like protein (ASC, also a CARD-containing protein, named PYCARD) to form inflammasome and promote the activation of caspase-1 8,14. In all cases, signals are transferred via CARD-CARD interaction among proteins. However, there are more than 30 CARD containing-proteins in mammals that are known to play a pivotal role in regulating inflammatory and apoptotic signaling. This raises question as to what determines which CARD-containing proteins are recruited by the RIG-I-like receptors. In general, CARD proteins such as caspases, NLRs, apoptotic protease activating facter-1 (Apaf-1), CARD 9/11, *etc* are thought to be involved in downstream signaling of RIG-I-like receptors^15^,^16^. However, the regulatory pathways and underlying mechanisms remain largely unknown. It is generally accepted that progressive antiviral activation of RIG-I-like receptors results in extensive cell injury that finally leads to apoptosis through a caspase-dependent apoptotic cell death mechanism^17^.

While most viruses induce dsRNA in the host cells, MDA5 and RIG-I recognize different types of dsRNA and exhibit different antiviral immunity to viruses^11^. RIG-I is essential for RNA viruses including paramyxoviruses, influenza virus and Japanese encephalitis virus, whereas MDA5 is critical for picornavirus detection. We postulate that the antiviral specificity of the RIG-I-like receptors could be determined by their dsRNA binding domains. It was thus possible to change the specificity of a RIG-I-like receptor by swapping its dsRNA binding domain with a proper dsRNA binding proteins. Under this assumption, it was also possible to manipulate downstream antiviral responses by swapping its CARD with a proper CARD-containing protein.

In this study, we examined a potential antiviral mechanism involving chimeric dsRNA-dependent RLR receptor by mimicking the dsRNA binding domain and the CARD domain. The dsRNA binding domain was selected from dsRNA-specific protein kinase (PKR) for its broad antiviral specificity. The CARD domain was chosen from Apaf-1 to directly activate caspase-9 and then apoptosis by bypassing the upstream antiviral pathways that might have already been blocked by viruses. We have previously demonstrated that the chimeric dsRNA-dependent RLR receptor, termed dsCARE in this study, provided high antiviral efficacy against infections from different types of viruses^18^ including the deadly Ebola virus^19^. Surprisingly, the chimeric RLR receptor had no effect on the IFN pathway and did not activate apoptosis; instead, it efficiently protected the uninfected cells by eliminating the infected cells via dsRNA-dependent RLR receptor-mediated necroptosis.

## Results

### An efficient antiviral strategy via chimeric dsRNA-dependent RLR receptor

**Figure 1:**
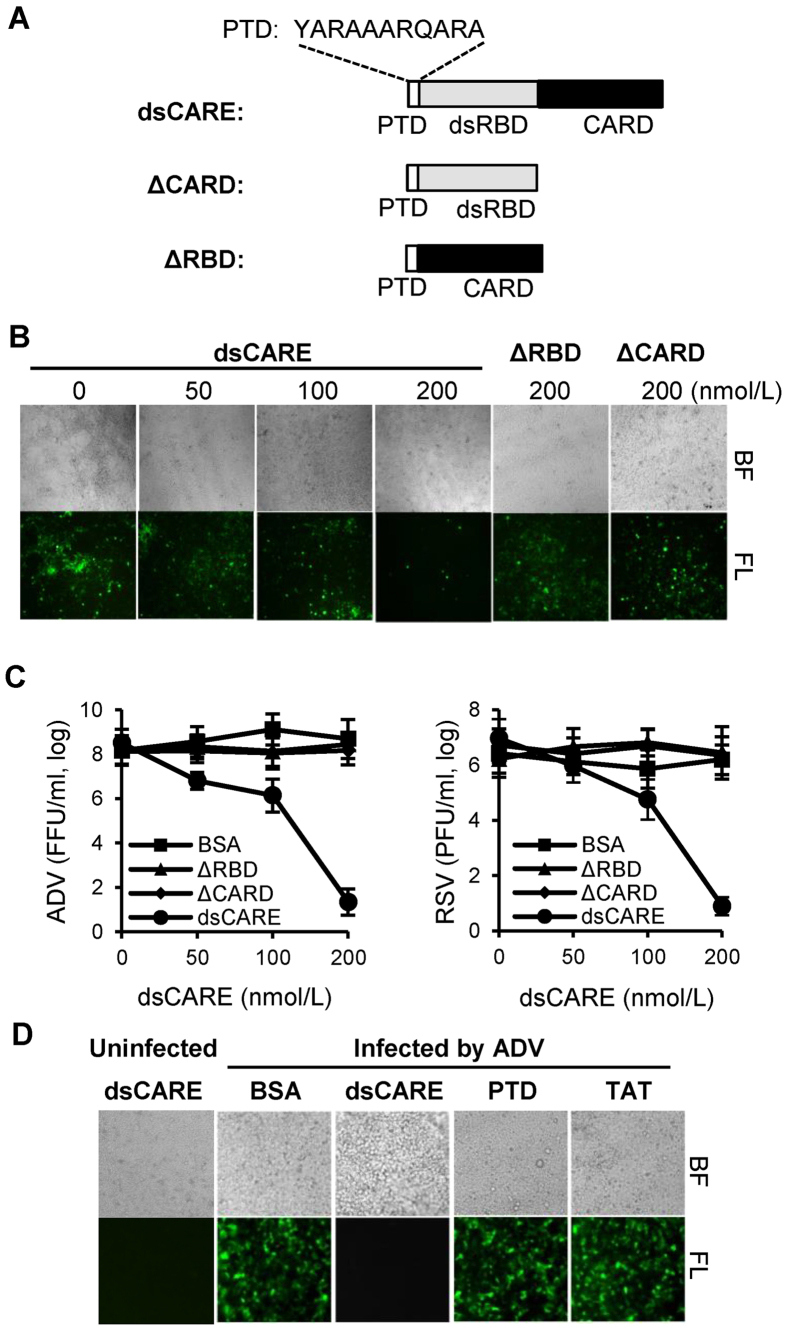
Inhibition of viral proliferation by recombinant dsCARE. (**A**) Domain structure of dsRNA-dependent caspase recruiter or dsCARE. It consists of a protein transduction domain (PTD), two repeats of dsRNA binding domain (dsRBD), and the caspase activation and recruitment domain (CARD). (**B**) Antiviral efficacy of dsCARE by fluorescent microscopy. Cells were pre-incubated with 0, 50, 100 and 200 nmol/L of dsCARE or 200 nmol/L of ΔRBD or ΔCARD for 1 hour. Photographs were taken at the 3rd day after ADV infection (MOI = 0.1). ΔRBD, dsCARE without dsRBD; ΔCARD, dsCARE without CARD. (**C**) Dose-dependent antiviral efficacy of dsCARE by virus titration. Cells were pre-incubated with 0, 50, 100, 200 nmol/L of dsCARE (BSA and ΔRBD as controls) for 1 hour and infected with ADV (MOI = 0.1) for 3 days (left panel). In parallel, cells were infected with RSV (MOI = 0.1) for 3 days (right panel). Virus titer was determined by means of 50% tissue culture infective dose (TCID50), and fluorescent-foci forming units (FFU) or plaque forming units (PFU) was calculated in Spearman-Karber Method. Log number of virus was plotted against dsCARE concentration (n = 6). (**D**) Antiviral efficacy of transduction domain PTD/TAT in comparison with dsCARE. Cells were pre-incubated with 200 nM BSA, dsCARE, PTD or TAT for 1 h. Photographs were taken at the 3rd day after ADV infection.

We previously designed a series of virus pattern recognition receptors containing dsRBD and CARD, termed dsCAREs, to mimic the structure of RLR receptor and demonstrated that dsCARE efficiently sensed virus-induced dsRNA in human cells and suppressed infection from different viral families^18^ including the Ebola virus^19^. To understand the underlying mechanism, the similar dsCARE and two truncated mutants lacking the CARD signal (ΔCARD) or the dsRNA binding domain (ΔRBD) were produced for this study (Fig. 1A). Briefly, the CARD was chosen from Apaf-1 to induce apoptosis of the virus-infected cells, two repeats of dsRBD from PKR were used to sense virus-induced dsRNA, the artificial protein transduction peptide (PTD4)^20^ was added at the N-termini to bring dsCARE through the cell membrane, and a His-tag was added to facilitate protein purification. The chimeric RLR receptor dsCARE was expressed in *E. coli* and purified to test their antiviral efficacy in cell culture. The chimeric dsCARE was added in the media and incubated with cells for absorbance before viral infection. Viruses from two representative viral families, adenovirus (ADV) from the dsDNA virus family and wild-type respiratory syncytial virus (RSV) from the negative-strand RNA virus family, were used to infect the human lung alveolar epithelial cell line A549 at low multiple of infection (MOI = 0.1). Because ADV expressed the green fluorescent protein (ADV-GFP), cells infected with ADV-GFP were monitored and photographed under fluorescent microscope. As shown in Fig. 1B, cells were infected with ADV-GFP in the absence of dsCARE; however, the infection was suppressed by dsCARE in a dose-dependent manner, indicating the antiviral activity of dsCARE. The antiviral activity of dsCARE was abolished when either CARD or dsRBD was deleted, suggesting the requirement of both dsRBD and CARD (Fig. 1B) for the antiviral activity of dsCARE. To further confirm the interaction between virus-induced dsRNA and dsCARE, dsCARE and the mutants lacking dsRBD (ΔRBD) or lacking CARD domain (ΔCARD) were compared to block the infection of ADV or RSV. Fluorescence-forming units (FFU) for ADV-GFP infection and plaque forming units (PFU) for RSV infection were quantified. Consistently, the titers of ADV and RSV were decreased by dsCARE in a dose-dependent manner, but not affected by ΔRBD or ΔCARD (Fig. 1C). Finally, to exclude the possible cytotoxicity of PTD4 and its effect on antiviral activity, the synthesized PTD4 and another transduction peptide TAT that was used as a control were tested and compared with dsCARE^20^. We found that only dsCARE had efficient antiviral activity whereas neither PTD nor TAT showed any effect (Fig. 1D). Thus, the chimeric dsCARE mimicking the RIG-I-like receptors (or chimeric RLR receptor) efficiently responded to and suppressed viral infection through the dsRBD and CARD dual functional domains.

### Negative regulation of the IFN pathway and positive regulation of pro-inflammatory IL-1β by the chimeric RLR receptor

**Figure 2:**
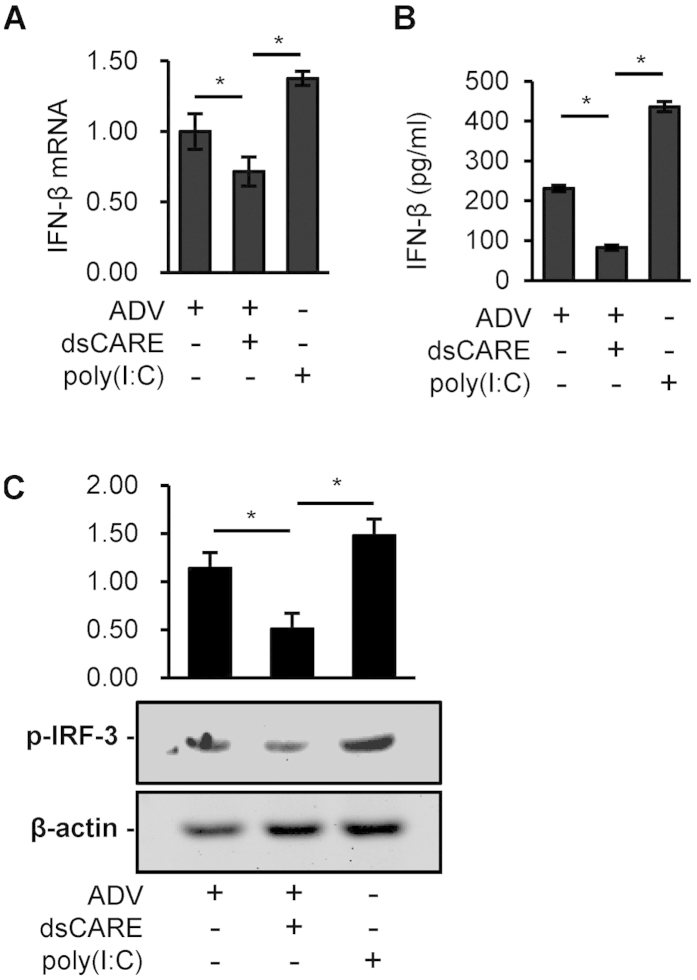
Negative regulation of the IFN pathway by the chimeric RLR receptor. (**A**) Cells were infected with ADV for 8 hours. After that, dsCARE was treated for 5 hours (200 nmol/L). BSA was used in ADV control. As a positive control, 10 μg/ml poly (I:C) was transfected into cells for 24 h. Total RNA was extracted and reverse transcribed. IFN-β mRNA was determined by quantitative PCR. *P < 0.05; data were means ± SD, n = 3. (**B**) The concentrations of IFN-β in the culture supernatants were measured by ELISA. *P < 0.05; data were means ± SD, n = 3. (**C**) Phospho-IRF-3 (p-IRF-3) was detected by western blotting in these groups and the intensities of the immunoreactive bands were quantified by densitometric scanning (n = 3). *P < 0.05; Data were means ± SD.

The IFN-β mediated antiviral pathway is known to play a central role in antiviral responses^9^. To evaluate whether dsCARE-dependent antiviral activity was related to the IFN pathway, we examined the transcription of IFN-β mRNA and the secretion of IFN-β protein in the cell culture. As a positive control, the synthetic dsRNA polyinosinic:polycytidylic acid (poly (I:C)) was transfected to activate the IFN pathway^9^. Adenovirus was found to induce IFN-β transcription (Fig. 2A) and enhance IFN-β secretion in the media (Fig. 2B) as in comparison with poly (I:C) transfection. However, addition of dsCARE suppressed virus-induced IFN-β expression at the levels of mRNA and protein (Fig. 2A,B). To confirm the negative regulation of the IFN-β pathway by dsCARE, we further examined the phosphorylation of IRF-3, the chief inducer of IFN-β. Consistently, both virus and poly (I:C) activated IRF-3 phosphorylation but virus-induced IRF-3 activation was suppressed when cells were pre-treated with dsCARE (Fig. 2C).

**Figure 3:**
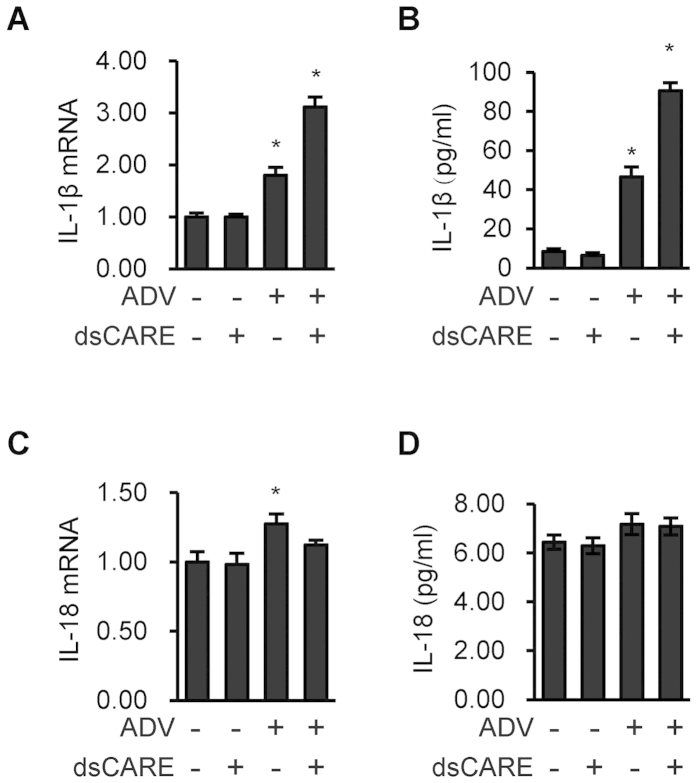
Increasing production of IL-1β by the chimeric RLR receptor. (**A**,**C**) Cells were infected with ADV for 8 hours and then treated with dsCARE at 200 nmol/L for 5 hours. BSA was used in blank control and ADV control. Total RNA was extracted and reverse transcribed. mRNA of IL-1β (**A**) and IL-18 (**C**) were quantified by quantitatie PCR. *P < 0.05, comparing to the ADV control; Data were means ± SD, n = 3. (**B**,**D**) The concentrations of IL-1β (**B**) and IL-18 (**D**) in the culture supernatants were measured by ELISA. *P < 0.05; comparing to the ADV control; data were means ± SD, n = 3.

In parallel with IFN type I expression, the formation and secretion of pro-inflammatory cytokines such as interleukin-1β (IL-1β) and IL-18 are other crucial downstream components of activated RLR pathway^7^,^8^,^21^. We thus examined mRNA expression and secretion of IL-1β and IL-18 using quantitative PCR and ELISA, respectively. Comparing to the blank control that was administrated with BSA (200 nmmol/L) and the negative control that was administrated with dsCARE (200 nmol/L) alone, we found that both ADV infection and dsCARE treatment after ADV infection led to increased transcription and secretion of IL-1β (Fig. 3A,B). This implies that dsCARE promotes the inflammation during antiviral progress. However, the expression of IL-18 was not apparently promoted by dsCARE when ADV infection increased transcription of IL-18 (Fig. 3C,D). These data suggested that dsCARE mediated an antiviral pathway that was different from the canonical IFN pathway; interestingly, dsCARE negatively regulated the IFN-β mediated antiviral pathway and promoted the expression and secretion of IL-1β.

### Antiviral prophylaxis through dsCARE-dependent infected cell death

**Figure 4:**
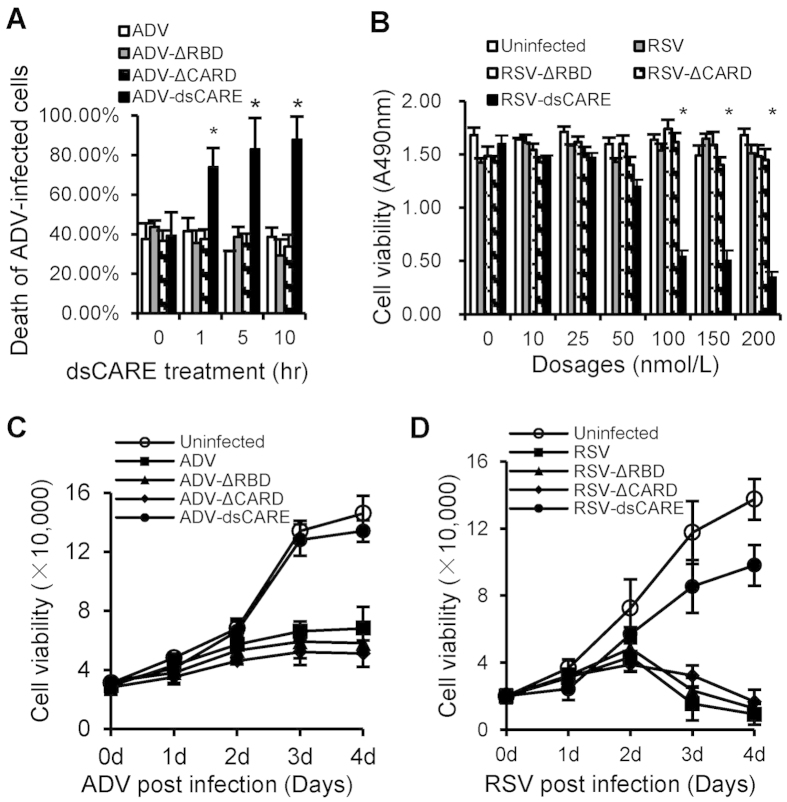
Antiviral prophylaxis through dsCARE-dependent infected cell death. (**A**) dsCARD-dependent infected cell death. Cells were uniformly infected by ADV (MOI = 10) for 1 day and then incubated with 200 nmol/L of dsCARE, ΔRBD, ΔCARD or BSA (control) for 0, 1, 5 and 10 hours. Dead cells (propidium iodide permeable) and ADV-infected cells (GFP positive) were determined by flow cytometry. Data were means ±  SD, n = 5, *P < 0.05. (**B**) Dose-dependent infected cell death. Cells were uniformly infected by RSV (MOI = 10) for 1 day before addition of 0, 10, 25, 50, 100, 150 and 200 nmol/L of dsCARE, ΔRBD, ΔCARD, or BSA (for RSV group or uninfected control) for 10 hours. Cell viability was determined by MTS assay. Data were means ± SD, n = 5, *P < 0.05. (**C**,**D**) dsCARE prevented cell lysis resulted from ADV (**C**) or RSV (**D**) infection. Cells were pre-treated with 200 nmol/L of dsCARE, ΔRBD, ΔCARD or BSA for 1 hour, and then infected with ADV or RSV (MOI = 0.1) for 0, 1, 2, 3, and 4 days. Cell viability was determined by trypan blue exclusion assay. Data were means ±  SD, n = 5, *P < 0.05.

To characterize the effect of dsCARE on virus-infected cells, A549 cells were infected with high titers of ADV-GFP (MOI = 10) to ensure uniform infection and cell death was examined by propidium iodide (PI) permeability using flow cytometry. The death of ADV-infected cells was detected after dsCARE was applied for 1, 5 and 10 hours and the number of death cells increased with time (Fig. 4A). However, ~40% cell death due to viral infection was observed when dsCARE was absent or truncated dsCARE was used, suggesting that dsCARE mediated virus-infected cell death. To further confirm dsCARE-dependent cell death, the wild type negative single-strand RNA virus, RSV, was used to infect A549 cells with high titers (MOI = 10). Cell death of uniformly RSV-infected cells was examined after dsCARE was applied for 10 hours. Similar to ADV infection, no death was observed after RSV infection in the absence of dsCARE or in the presence of truncated dsCARE (Fig. 4B); however, cell death took place when dsCARE was applied, starting at 50 nmol/L, and the number of dead cells increased with dsCARE in a dose-dependent manner. Nearly 80% of RSV-infected cells underwent cell death with 200 nmol/L dsCARE. In contrast, no cell damage was observed when the truncated mutants, ΔRBD and ΔCARD, were used. These observations suggested that virus-infected cell death was dependent on dsCARE and was activated by dsRNA-dependent CARD signaling.

To quantify the protective effect of dsCARE on uninfected cells, A549 cells were pre-treated with dsCARE prior to infection with a low titer (MOI = 0.1) of ADV (Fig. 4C) or RSV (Fig. 4D) to ensure only a small population (<10%) of cells was initially infected. At the following days post infection when the amplified viruses would spread and virus-induced cytolytic cell death would be observed, cell viability was examined by trypan blue exclusion assay. In the absence of dsCARE or in the presence of truncated dsCARE, cell viability started to decline at the 2^nd^ day post infection, reaching to ~10% in comparison with the control cells at the 4^th^ day post infection (Fig. 4C,D). In the presence of dsCARE, however, cells were still viable in a comparative pattern as the uninfected control. Thus, dsCARE protected the cell population from infection by eliminating the initially infected cells. Cumulatively, these data suggested that viruses likely induced the expression of long stretches of dsRNA in infected cells, which activated a dsRNA-dependent CARD signaling through the chimeric RLR receptor, leading to death of the infected cells. In contrast, dsCARE had no effect on uninfected cells, supporting the concept that no long stretches of dsRNA exist in cells under healthy conditions.

### Apoptosis is not activated in dsCARE-dependent infected cell death

**Figure 5:**
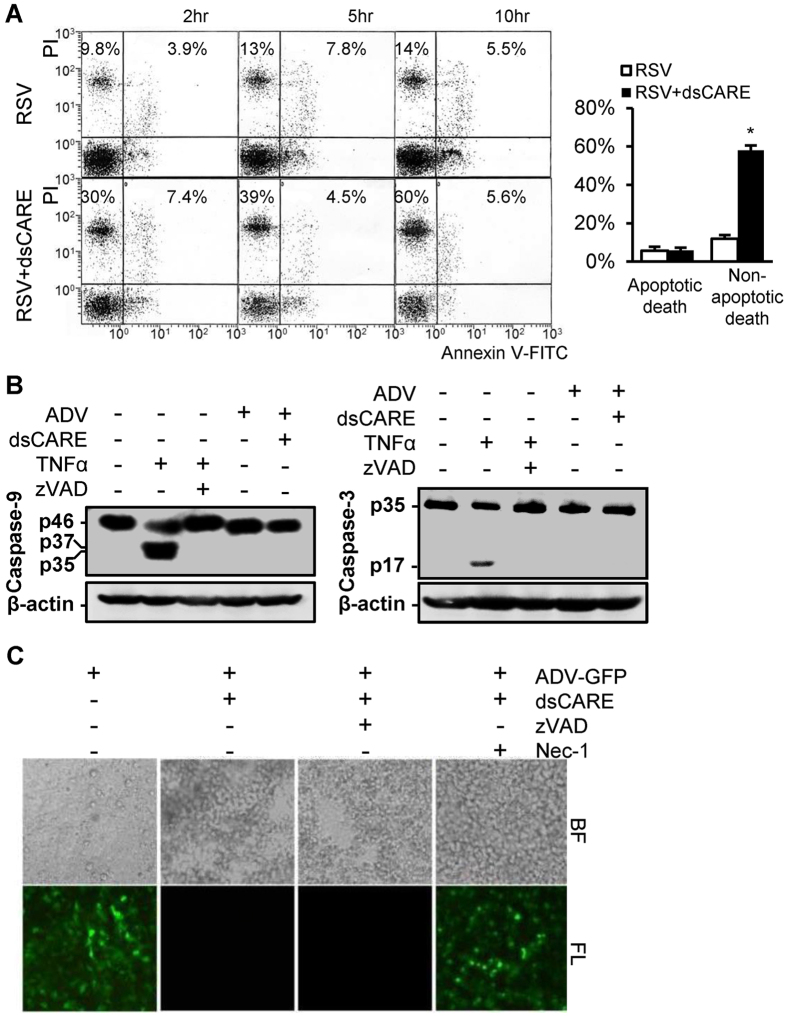
Exclusion of apoptosis for dsCARE-dependent infected cell death. (**A**) Apoptotic and non-apoptotic cell death of uniformly RSV-infected cells by flow cytometry. Cells were uniformly infected with RSV (MOI = 10) for 1 day and then exposed to 200 nmol/L dsCARE for additional 2, 5 and 10 hours. Cell death was determined by flow cytometry. Apoptosis (double positive) and non-apoptotic death (PI positive) of exposed cells in 10 hours group were quantified and statistically tested (right panel). Data were means ±  SD, n = 3, *P < 0.05. (**B**) Western blotting of apoptosis-specific cleavage of caspase-9 (left panel) and -3 (right panel) using the antibodies against human caspase-9/-3. Cells were infected with ADV in the absence or presence of dsCARE. TNF-α was used as the apoptotic control; TNF-α with zVAD pre-treatment was used as the necrotic control. In the left panel: p46, pro-caspase-9; p37 and p35, the cleavage products. In the right panel: p35, caspase-3; p17, the cleavage product. (**C**) Inhibition of dsCARE-dependent infected cell death by zVAD or Nec-1. ADV-infected cells were pre-treated with 200 nmol/L dsCARE in the presence of zVAD (50 μmol/L) or Nec-1 (30 μmol/L) for 2 hours. Photographs were taken 24 hours post-infection under bright field (BL) or fluorescent (FL) microscope.

As the CARD in the chimeric RLR receptor was from Apaf-1 and designed to activate caspase-9-mediated apoptosis^22^,^23^, we next examined whether apoptosis was activated in dsCARE-dependent infected cell death. Several canonical markers of apoptosis were examined. Firstly, phosphatidylserine exposure is known as a typical apoptotic measure detected as Annexin V positive by flow cytometry^24^. Thus we examined RSV-infected cells (MOI = 10) to determine apoptotic (Annexin V-FITC positive) and non-apoptotic (propidiumiodide positive) cell death in the presence or absence of dsCARE (Fig. 5A). Surprisingly, the extent of apoptotic cell death did not change with or without dsCARE (average of 5.8% and 5.7%, *t*-test, *P* = 0.49). However, non-apoptotic cell death was significantly increased by dsCARE, which was 9.8%, 13% and 14% in the absence of dsCARE and 30%, 39% and 60% with dsCARE treatment for 2, 5 and 10 hours respectively, suggesting a non-apoptotic mechanism. To confirm this unexpected observation, apoptosis-specific cleavage of caspases was examined. Here, TNF-α was used as the positive control, because it is known to induce apoptosis by the cleavage of caspase-9 and -3 25, and the cleavage is suppressed by a cell permeable pan-caspase inhibitor zVAD^26^,^27^ (Fig. 5B). When ADV-infected A549 cells (MOI = 10) with or without dsCARE treatment were examined, the cleavage of caspase-9 and -3 was not prominent, excluding the activation of caspases-9 and -3. Furthermore, the caspase inhibitor zVAD did not affect the antiviral function of dsCARE against ADV infection (MOI = 0.1) (Fig. 5C). Taking together, we concluded that apoptosis was not the prominent mechanism and thus a non-apoptotic pathway appeared to be accountable for dsCARE-dependent infected cell death.

### Transmission electron micrograph indicates dsCARE-dependent necrotic death of virus-infected cells

**Figure 6:**
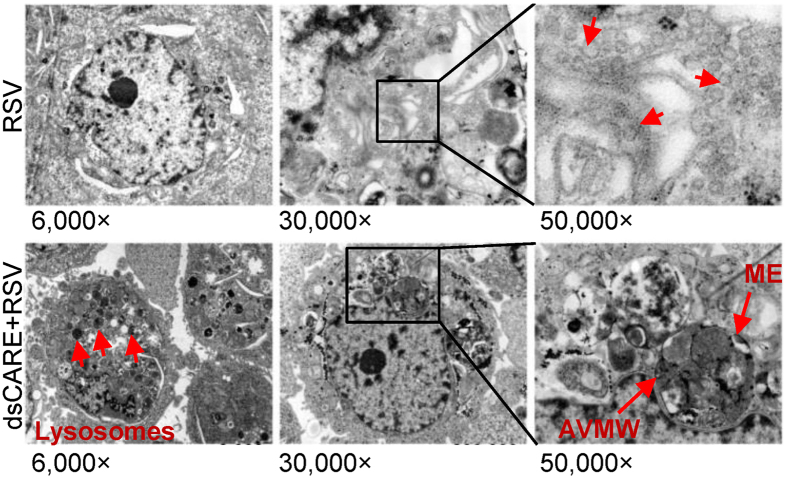
Transmission electron micrograph indicates dsCARE-dependent necroptosis of infected cells. RSV-infected cells (MOI = 10) with/without dsCARE treatment were fixed for transmission electron microscopy. ME, mitochondria embedding; AVMW, autophagy vacuoles containing membranous whorls.

Morphologic analysis of dsCARE-dependent cell death was performed using transmission electron microscopy. For RSV-infected cells, cell membrane was intact (Fig. 6, 6000×), organelles were normal (30 000×), and a typical viral particles were observed (cutout view); while addition of dsCARE post uniform infection resulted in necrotic features characterized by loss of cell membrane integrity, lysosome formation, mitochondrial autophagy and cell lysis (Fig. 6). In addition, mitochondrial embedding (ME) and autophagy vacuoles containing membranous whorls (AVMW) were observed, that were consistent with non-apoptotic necrotic cell death. In line with these findings, necrostatin-1 (Nec-1), a specific inhibitor for programmed necrosis or necroptosis^26^,^28^, blocked the antiviral effect of dsCARE whereas the apoptosis-specific caspase inhibitor zVAD did not (Fig. 5C). Those suggested that dsCARE eliminated infected cells by activating a dsRNA-dependent necroptosis pathway rather than apoptosis.

### The chimeric RLR receptor mediated RIP1, RIP3 and cathepsin D-dependent necroptosis

**Figure 7:**
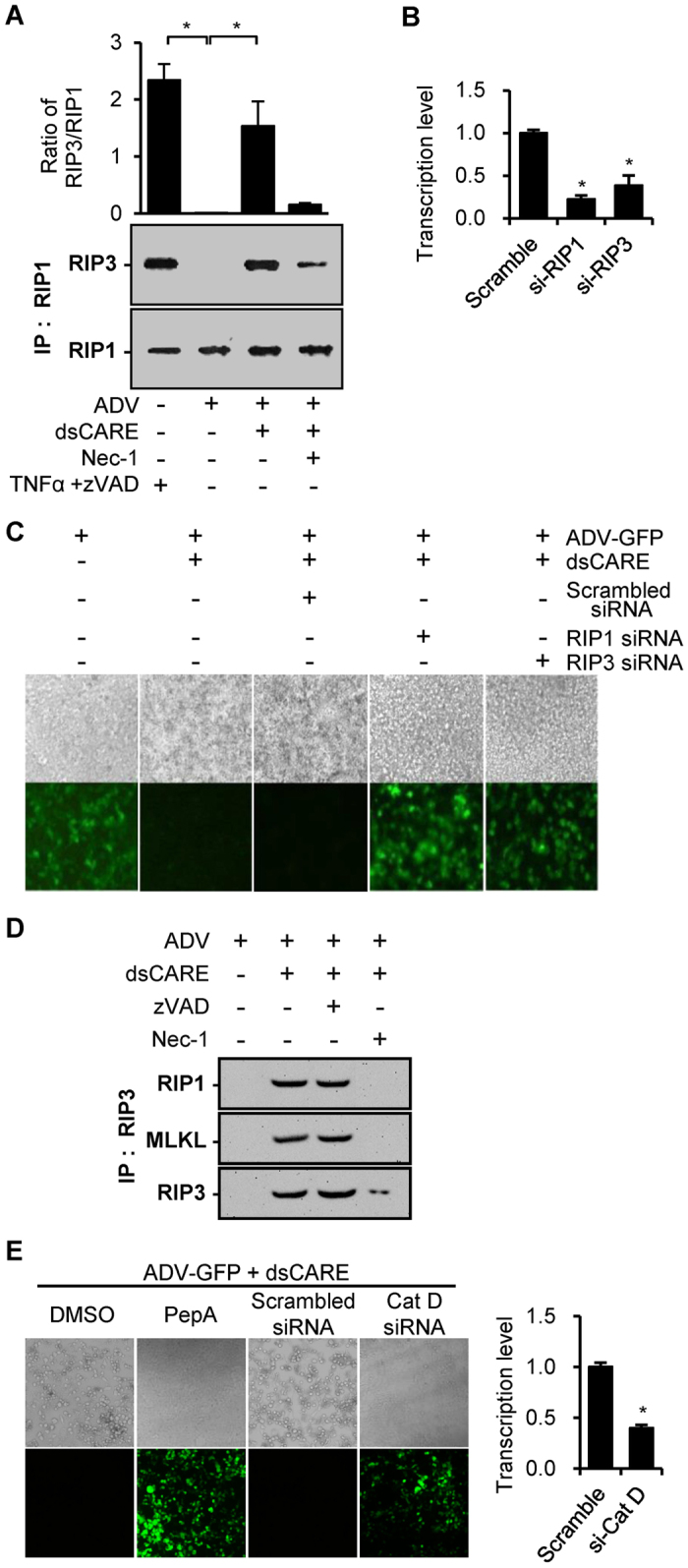
Necrosome mediates dsCARE-dependent infected cell death. (**A**) Detection of the RIP1/RIP3 necrosome by immunoprecipitation followed by Western blotting. ADV-infected cells (MOI = 10) were treated with dsCARE with/without Nec-1. The necrosome immunoprecipitated with the anti-RIP1 antibody was detected by Western blotting with anti-RIP1 and RIP3 antibodies. The ratio between RIP1 and RIP3 was quantified by densitometric scanning. TNF-α + zVAD: positive control for necrosome. Data were means ±  SD; *with/without dsCARE, with/without Nec-1, *P* < 0.05; n = 3. **(B**,**C**) Cells were transfected with scrambled siRNA, RIP1 siRNA or RIP3 siRNA for 24 hours and mRNA levels were determined by quantitative PCR (**B**) (n = 3, *P < 0.05). The transfected cells were then infected with ADV (MOI = 0.1) with/without dsCARE. Photographs were taken 2 days after infection (**C**). (**D**) Recruitment of MLKL in the RIP3 necrosome by Immunoprecipitation-Western blotting. Cell lysates from ADV-infected cells (MOI = 10) with indicated treatments were immunoprecipitated with the anti-RIP3 antibody and the necrosome were examined by Western blotting using the anti-RIP1, anti-RIP3 or anti-MLKL antibodies. (**E**) Cells were treated with PepA (or DMSO as the negative control) for 2 hours or transfected with cathepsin D siRNA (or scrambled siRNA as the negative control) for 24 hours. The mRNA level of cathepsin D was determined by quantitative PCR. *P < 0.05, n = 3. Treated cells were then infected with ADV (MOI = 0.1). Photographs were taken 2 days after infection.

The elicitation and execution of programmed cell death is controlled by a dynamic network between the apoptotic and necroptotic pathways with typical features such as caspase activation and necrosome formation, respectively^23^,^29^,^30^,^31^. To confirm that the chimeric RLR receptor induced necroptosis, we examined necrosome formation of the receptor-interacting protein RIP1 and RIP3, the essential complex of the serine/threonine kinases for necroptosis^26^,^29^,^32^. RIP1 is known as a component specific for TNF-α receptor-associated apoptosis, deemed as a “switch” between apoptosis and necroptosis^23^,^29^, while RIP3 is the core component of necrosome in necroptosis^26^,^29^,^33^. The necrosome complex was then immunoprecipitated with the anti-RIP1 antibody and RIP3 were detected by Western blotting using the antibodies against RIP1 and RIP3. The model of TNF-α/zVAD induced necroptosis was used as the positive control, in which the RIP1-RIP3 necrosome was formed when TNF-α-induced apoptosis was blocked by zVAD^26^,^32^. Remarkably, RIP3 was recruited to RIP1 in positive control and in ADV-infected cells that were only treated by dsCARE, and the recruited RIP3 was dramatically reduced in cells with the necroptosis inhibitor Nec-1 (Fig. 7A). In the antiviral assay, the essential requirement for RIP1 and RIP3 was confirmed by knockdown of RIP1 or RIP3 expression using RIP1 or RIP3 specific siRNA. Transfection of either RIP1 or RIP3 siRNA blocked the antiviral effect of dsCARE against ADV infection whereas transfection of scrambled siRNA had no effect (Fig. 7B,C). Recent studies have shown that RIP3 is required for the recruitment of downstream mediators of necroptosis^34^. The mixed lineage kinase domain like (MLKL) protein is one of the mediators^33^,^35^ and an indispensable substrate of RIP3^33^,^36^. To further confirm necroptosis activation, the recruitment of MLKL in the RIP3 necrosome in dsCARE-dependent death of ADV-infected cells was examined by immunoprecipitation with the anti-RIP3 antibody followed by Western blotting with the anti-RIP1 and anti-MLKL antibody. Indeed, MLKL in ADV-infected cells was detected only when dsCARE was added and blocked when both Nec-1 and dsCARE were added (Fig. 7D). To verify whether the chimeric RLR dsCARE mediated necroptosis via the same path as the natural RLR pathway, we used the chemical inhibitor pepstatin A (PepA)^37^ and the specific siRNA of cathepsin D to block RLR-mediated necroptosis. In comparison with respective controls, we found inactivation or knockdown of cathepsin D resulted in loss of antiviral function for dsCARE (Fig. 7E). These findings suggested that the chimeric RLR dsCARE induced necroptosis via natural necroptotic pathway of RLRs to prevent viral infection.

## Discussion

In this study, we demonstrated that dsCARE mimicking the RIG-I like receptor (RLR) family prohibited viral infection by eliminating infected cells from the innate immune system via programmed cell necrosis or necroptosis, an important innate immune response of mammals uncovered within the last decade. Our data showed that the chimeric RLR receptor was capable of detecting virus-associated dsRNA and activating necroptosome that consisted of RIP1, RIP3 and MLKL in virus-infected cells via lysosomal enzyme cathepsin D in addition to promoting the expression of inflammatory cytokine IL-1β but inactivating the well documented IFN pathway.

Although it remains to be further understood how host cells initiate necroptosis to defend against infection, activation of necroptotic pathways has been documented in infections induced by vaccinia virus, HIV-1, and cytomegalovirus^26^,^38^,^39^,^40^. We provide evidence that dsRNA-dependent RLR receptors are the first line of innate defense mechanism that is capable of activating the necroptotic pathway during viral infection. This may be of great importance as the RLR family is the solely cytoplasmic dsRNA-dependent CARD-containing receptor of mammalian innate immune system. Dual mechanisms appear to be involved as its dsRBD domain senses virus-associated dsRNA whereas the CARD domain activates IFN antiviral pathway and eventually eliminates severely infected cells by programmed cell death^8^,^41^. Usually, apoptosis is considered as the final cleanup mechanism following the production of IFN and pro-inflammatory cytokines for dsRNA-induced RLR signaling in the severely infected cells, requiring the cleavage of pro-caspase-9/-3 followed by a caspase-dependent cascade. However, we demonstrated that the chimeric RLR receptor dsCARE did not activate the IFN pathway (Fig. 2). This observation was supported by canonical cell death analysis that excluded the involvement of apoptosis for the infected cell death (Figs 5, 6, 7). Unlike apoptosis, necroptosis is characterized by the loss of membrane integrity, swelling of cell organelles in dying cells, the lack of nuclear condensation, and cellular lysis^23^,^29^. Necrotic changes that were detected by morphologic assessment, phosphatidylserine exposure, caspase cleavage and specific caspase inhibition provided compelling evidence and consistently suggested that the chimeric RLR receptor activated necroptosis of virus-infected cells.

Traditionally, necroptosis is referred to a downstream ending mechanism of tumor necrosis factor receptor (TNFR) and TLR3 pathways, which is known to be a RIP3-dependent but caspase-independent process^26^,^33^. TNFR-mediated necroptosis is induced by caspase-specific inhibitor zVAD and suppressed by necroptosis specific inhibitor Nec-1 28,42. This is consistent with our observations (Figs 5 and 7), showing that the chimeric RLR receptor-mediated antiviral activity was suppressed by Nec-1 but was not affected by zVAD. Typically, a necrosome is formed between RIP1 and RIP3 that recruits the downstream MLKL as an indispensable component to execute necroptosis^36^. Accordingly, we demonstrated that dsCARE-mediated infected cell death was featured with the inactivation of caspase-9/-3 and IFN-β pathway and the activation of the RIP1-RIP3-MLKL necrosome. Consistent with necroptotic phenomenon, zVAD had no adverse effect on dsCARE, whereas Nec-1 impeded its antiviral effects. In TNFR and TLRs pathways, although RIP1 is a required factor to switch apoptosis to necroptosis^33^,^43^; RIP3 initiates necroptosis even when RIP1 is depleted^34^. Our data provided evidence that both RIP1 and RIP3 were involved and were essential to dsCARE-mediated necroptosis. While membrane receptors mediated necroptosis such as TNFR and TLRs pathways are well documented, underlying mechanisms of RLRs induced necroptosis are poorly understood. It was recently reported that dsRNA could elicit necroptosis via RLRs by activating a lysosomal enzyme, cathepsin D^32^,^37^. Consistently, we observed the emerging of large amount of lysosomes when dsCARE was applied to infected cells under transmission microscope (Fig. 6, 6000×, red arrows) and found that dsCARE did not function with the blockade of cathepsin D (Fig. 7E). These findings suggest that the chimeric dsRNA-dependent receptor dsCARE acts as a RLR receptor and evades the RLR-mediated apoptosis.

Necroptosis was originally discovered in Apaf-1-mutant mice, in which the key adaptor protein Apaf-1 that mediates caspase-9 activation is blocked^44^,^45^,^46^. Both Apaf-1 and caspase-9 are CARD proteins and the homophilic CARD/CARD interaction is shown to activate their function and lead to apoptosis^47^,^48^. When the CARD interaction between Apaf-1 and caspase-9 is blocked, TNF-α receptor-associated apoptosis is rapidly switched to necroptosis^46^. The dsRNA-dependent RLR receptors used in this study are also CARD proteins and their interaction with the CARD-containing adaptor MAVS crucial to the induction of interferon in response to viral infection^7^. Initially, we reasoned that, by swapping the RLR receptor CARD with the Apaf-1 CARD, the chimeric RLR would directly activate Apaf-1/caspase-9, bypassing the common IFN pathway. Theoretically, apoptosis is anticipated, which is initiated and regulated by the CARD proteins, including Apaf-1, caspases, BCL-2 family^16^,^41^. The apoptosome consisting of cytochrome *C*, Apaf-1 and pro-caspase-9 is formed to cleave caspase-3 and execute apoptosis^41^,^49^. Unexpectedly, our data indicated that the CARD domain from Apaf-1, when fused with the dsRBD from PKR, led to the formation of necrosome and execution of necroptosis in virus-infected cells. This observation suggests that the chimeric RLR receptor or dsCARE suppressed the Apaf-1 function and blocked activation of apoptosis probably in a similar way as in Apaf-1-deficient cells^44^,^46^. Thus, instead of establishing a shortcut to activate Apaf-1-mediated apoptosis, the chimeric RLR receptor rewired the dsRNA-induced IFN antiviral pathway and apoptosis with Apaf-1-deficiency-induced necroptosis. Nevertheless, our data also suggest that the CARD domain in the RLR receptors is functionally independent and can be replaced with other CARDs of CARD-containing factors to constitute novel dsRNA-dependent RLR receptors with different innate immune responses.

Because most viruses induce dsRNA in mammalian cells during infection^5^,^6^, long stretches of dsRNA is thought to have evolved as a virus-associated molecular pattern^7^. RIG-I and MDA5 are known dsRNA-dependent receptors that have different preferences for dsRNA and virus-associated pattern selection^22^. In comparison, PKR is involved in the innate antiviral response against infection of a wide spectrum of viruses^50^. In our study, the dsRBD from PKR was chosen in the chimeric RLR receptor to sense both viral and cellular dsRNA induced by viral infection and provoke dsRNA-dependent antiviral immunity with a wider coverage. In our previous and present studies, several dsRBDs from different proteins including PKR, ADAR1, PACT, and NF90 have been tested to constitute novel chimeric dsRNA-dependent RLR receptors^18^. Among them, the chimera with dsRBD from PKR and CARD from Apaf-1 was the most efficient antiviral RLR receptor largely due to rapid necroptotic properties and the dsRBD from PKR capable of sensing a wide spectrum of viruses. Cumulatively, our current and previous studies provide evidence that the chimeric RLR receptor exhibited efficient anti-infection properties against wide range of DNA and RNA viruses including ADV, RSV, VSV, and Ebola virus^18^,^19^.

In conclusion, our study suggests that the dsRNA-dependent chimeric RLR receptor activates necroptosis in viral infection to selectively eliminate virus-infected cells by targeting virus-associated dsRNA. As most viruses induce dsRNA in the host cells, the chimeric RLR receptor-mediated necroptosis could be developed as a broad antiviral mechanism to prevent infections from a wide spectrum of viruses. Our findings warrant further evaluation of the prophylactic and therapeutic potentials of the chimeric RLR receptors against viral infections including Ebola virus, viruses with unknown background under emergency conditions, as well as newly emerged or frequently mutated viruses. In addition, the chimeric RLR receptor provides an effective approach to explore dsRNA-induced innate immune mechanisms during infection.

## Methods

### Cells, viruses and reagents

Human embryonic kidney cells (HEK293T, cat #SCSP-502), human lung alveolar epithelial cells (A549, cat #TCHu150) and wild type respiratory syncytial virus (RSV) long strain (Cat #GDV052) were purchased from Cell Bank of Chinese Academy of Sciences (CCTCC). Recombinant Adenovirus (ADV) that is packaged in HEK293T cells with the plasmid pSG500-GFP and pBGHlox and that could be replicated in tumor cells was provided by Sinogenomax Inc., Beijing, China. Primary antibody against forphospho-IRF-3 (S386) was purchased from Abcam. Primary antibodies for caspase-9 and -3 were purchased from Cell Signaling Technology. Primary antibodies for 6×His-tag, RSV fusion protein, RSV glycoprotein G, RIP1, RIP3 and the mixed lineage kinase domain like protein (MLKL), and horseradish peroxidase or fluorescent labeled secondary antibodies of goat anti-mouse IgG, goat anti-rabbit IgG and rabbit anti-goat IgG were purchased from Santa Cruz Biotechnology. Caspase inhibitor zVAD-fmk (zVAD), necroptosis blocking agent necrostatin-1-fmk (Nec-1) and cathepsin D inhibitor pepstatin A (PepA) were purchased from Santa Cruz Biotechnology, TNF-α and Bovine serum albumin (BSA) from Sigma-Aldrich, and propidium iodide (PI) from Beyotime Institute of Biotechnology, China.

### Plasmids and constructs

The plasmids encoding for dsCARE and its truncated mutant were consisted of 1) the protein transduction domain (PTD)^20^, 2) two repeats of dsRNA binding domain (dsRBD) from dsRNA-dependent protein kinase (PKR), and 3) the caspase recruitment domain (CARD) from apoptotic peptidase activating factor 1 (Apaf-1)^18^. The DNA for PTD was made by two oligonucleotides, AGCTTGGATCCTACGCCCGTGCCGCCGCCCGTCAGGCCCGTGCCAGTGGT and CCATCTCGAGACCACTGGCACGGGCCTG, which were annealed and filled by PCR. The DNAs for dsRBD and CARD were amplified from human PKR cDNA (GenBank BC093676) and human Apaf-1 cDNA (GenBank DN998849), respectively. The full-length dsCARE and its truncated mutants were sequentially linked with proper restriction cleavage sites and cloned into the pRSET B expression vector (Invitrogen) for recombinant protein production in bacteria. All DNAs were confirmed by restriction endonuclease digestion and sequencing.

### dsCARE protein expression, purification and refolding

The recombinant protein of full-length dsCARE and the truncated mutants were transformed into *E. coli* strain BL21 (DE3). Single colonies were selected to grow in Luria-Bertani medium with 100 μg/ml of ampicillin. Bacteria were collected by centrifugation and re-suspended in lysis buffer (PBS containing 0.1% NP-40, pH 7.4). After sonication, inclusion bodies were collected and dissolved in the lysis buffer containing 8 mol/L urea. Supernatant was collected by centrifugation and applied to nickel resin column (GE Healthcare) following the manufacture’s protocol. The bound proteins were washed with the lysis buffers with decreasing concentration of urea, allowing the proteins to refold. The refolded dsCARE was eluted with 300 mmol/L imidazole. Its concentration was determined by bicinchoninic acid (BCA) assay (Thermo), and quality confirmed on 15% SDS-PAGE. Endotoxin was removed using endotoxin removal resin (Thermo).

### Adenovirus infection, titration and antiviral efficacy

A549 cells were cultured in RPMI 1640 medium (Hyclone), supplemented with 10% fetal bovine serum (FBS; Gibco) and 1% penicillin/streptomycin (Life Technologies). Cells at 60~70% confluences were infected with Adenovirus (ADV) at a low titer (MOI = 0.1) for 2 hours. Cells were refreshed with 2% FBS medium containing dsCARE at indicated concentrations and continued cultivating at 37 °C with 5% CO_2,_ until infected cells predominated by florescent microscopy. To evaluate antiviral efficacy, cells were re-suspended in the same medium and diluted at 10-fold series ranging from 10^−1^ to 10^−14^ in 96-well plates. Then green fluorescent protein (GFP)-positive wells were counted under florescent microscopy. The fluorescent-foci forming units (FFU) was calculated with the Spearman-Karber Method. Antiviral efficacy was determined by the relative virus titers with and without dsCARE or the mutants.

### Respiratory syncytial virus (RSV) infection, titration and antiviral efficacy

Semi-confluent A549 cells were infected with RSV at a low titer (MOI = 0.1) for 2 hours. Cells were refreshed with 2% FBS medium containing dsCARE at indicated concentrations and continue cultivating at 37 °C with 5% CO_2_, until syncytial cytopathic effect was widely observed under bright field of microscope. To evaluate antiviral efficacy, cells were freeze-thawed in the medium three times to release virus into supernatant, and then the supernatant was passed through a 0.2 μm filter. The supernatant was diluted in a 10-fold series and added in 96-well plates coating with the anti-RSV antibody. Virus was detected by sandwich ELISA (plates were coated with anti-fusion protein antibody and viral particles were detected with anti-glycoprotein G antibody) and Plaque Forming Unit (PFU) was calculated with the Spearman-Karber Method.

### Cell viability, growth inhibition and death

Usually high titers of virus were used to achieve a uniform cell infection so that the effect of dsCARE on cell viability, growth inhibition and death can be evaluated. Typically, cells were seeded in 6-well plates for 12 hours at 1.5×10^5^ cells/well. A high titer of ADV or RSV (MOI = 10) was added to the medium for viral attachment; different amounts of dsCARE were added at 8 hours after viruses were uniformly infected. To determine cell viability with the one-step 3-(4,5-dimethyl-2-thiazolyl)-2,5-diphenyl-2-H-tetrazolium bromide (MTS) assay (Promega), the mixed MTS solution was added into each well and absorption at 490 nm determined by NanoDrop (Thermo) according to the manufacturer’s protocol. To determine cell death with trypan blue exclusion assay, cells were collected and mixed with an equal volume of 0.4% trypan blue (Sigma-Aldrich); the stained dead cells and unstained viable cells were counted using the hemocytometer chamber under microscope. To determine apoptosis with flow cytometry assay, cells were collected, washed with PBS three times and incubated with fluorescein isothiocyanate (FITC)-conjugated Annexin V or Propidium Iodide (PI) for 30 min. Annexin V-positive and PI-positive cells were determined by flow cytometry (BD Biosciences).

### Assays for IFN-β and inflammatory cytokines

mRNA transcription was evaluated with quantitative PCR. Total RNA was isolated with RNAiso reagent (Clontech) and reverse transcribed with RT reagent Kit with gDNA Eraser (Clontech). Quantitative PCR analysis was performed using a 7500 fast Real-Time PCR System (Applied Biosystems) with RT Master Mix (Clontech) and following primers: human IFN-β, 5′-ACTGCCTCAAGGACAGGATG-3′ and 5′-AGCCAGGAGGTTCTCAACAA-3′; IL-1β, 5′-TTAAAGCCCGCCTGACAGA-3′ and 5′-GCGAATGACAGAGGGTTTCTTAG-3′; IL-18, 5′-ATCACTTGCACTCCGGAGGTA-3′ and 5′-AGAGCGCAATGGTGCAATC-3′; β-actin, 5′-GCTCCTCCTGAGCGCAAG-3′ and 5′-CATCTGCTGGAAGGTGGACA-3′. ELISA for IFN-β, IL-1β and IL-18 (R&D systems) was performed in accordance with the manufacturer’s instruction.

### Transfection and gene knockdown

For poly (I:C) transfection, cells were transfected at ~70% confluence with the final concentration of 50 μg/ml poly (I:C) using Lipfectamine 2000 (Invitrogen) following the manufacturer’s protocol. For gene knockdown, 150 nmol/L of double-stranded RIP1 siRNA oligonucleotides 5′-UGCUCUUCAUUAUUCAGUUUGCUCCAC-3′, RIP3 siRNA oligonucleotides 5′-UAACUUGACGCACGACAUCAGGCUG-3′, or scrambled siRNA oligonucleotides 5′-UUCUCCGAACGUGUCACGU-3′^26^ were chemically synthesized (Beijing AuGCT DNA-SYN Biotechnology, China), and cathepsin D siRNA was purchased from Santa Cruz Biotechnology. The siRNA was transfected using Lipfectamine 2000. Quantitative PCR was performed to identify the knockdown efficacy using the following primers: RIP1, 5′-TGGGAAAGCACTGGAAAAAC-3′ and 5′-GTCGATCCTGGAACACTGGT-3′; RIP3, 5′-TTTGGCCTGTCCACATTTCAG-3′ and 5′-GGTTGGCAACTCAACTTCTCTT-3′; Cathepsin D, 5′-TGCTCAAGAACTACATGGACGC-3′ and 5′-CGAAGACGACTGTGAAGCACT-3′. At 36 hours post-transfection, cells were infected with ADV (MOI = 0.1) in the presence or absence of dsCARE. The antiviral efficacy of dsCARE was examined by fluorescent microscopy.

### Western blotting and immunoprecipitation

Cells were washed with PBS and lysed in the RIPA buffer (Beyotime Institute of Biotechnology, China) supplemented with protease inhibitor cocktails (Beijing CoWin Bioscience, China). The protein concentrations of whole cell extracts were determined by BCA assay (Thermo). The proteins were resolved on 12% SDS-PAGE and analyzed by Western blotting with antibodies against caspase-3 (1:200), caspase-9 (1:500), 6×His-tag (1:500), p-IRF-3 (1:1000), RIP1(1:400), RIP3 (1:500), MLKL (1:800), or β-actin (1:1 000). For immunoprecipitation, the anti-RIP1 or anti-RIP3 antibody was used to precipitate the complex using the Immunoprecipitation kit (Thermo) following the manufacturer’s protocol. The protein complex was resolved on 12% SDS-PAGE and analyzed by immune blotting using antibodies against RIP1, RIP3 or MLKL. The membrane was stained with proper secondary antibodies conjugated with horseradish peroxidase and detected by electro-generated chemiluminescence imager (UVP BioImaging System).

### Transmission electron microscopy

Monolayer of infected cells with/without dsCARE were washed with PBS three times and centrifuged for 20 minutes at 1 000 g. Cell pellets were fixed in 4% paraformaldehyde in Hank’s solution, washed 3 times in Hank’s solution, post-fixed in 1% osmium tetroxide, dehydrated in ethanol and acetone, and embedded in epon araldite. Ultrathin sectioning was performed using a diamond knife. The sections were contrasted by uranyl acetate and lead citrate and examined with Jem 1400 transmission electron microscope (JEOL) at 80 kV. Digital photos were collected by a side-mounted Veleta digital camera (SIS). All reagents were obtained from Structure Probe.

### Statistical analysis

Statistical significance was assessed by Student’s *t*-test for two samples, and one-way ANOVA followed by Dunnett’s post hoc test for multiple comparisons. The data is presented as mean ± standard deviation (SD) of the mean. All experiments were independently repeated three times. Significance was determined at *P *≤ 0.05.

## Additional Information

**How to cite this article**: Wang, W. *et al.* Activation of innate antiviral immune response via double-stranded RNA-dependent RLR receptor-mediated necroptosis. *Sci. Rep.* **6**, 22550; doi: 10.1038/srep22550 (2016).
